# Silenced Knowing: An Intersectional Framework for Exploring Black Women's Health and Diasporic Identities

**DOI:** 10.3389/fsoc.2020.00001

**Published:** 2020-02-07

**Authors:** Laura Serrant

**Affiliations:** Faculty of Health, Psychology and Social Care, Manchester Metropolitan University, Manchester, United Kingdom

**Keywords:** intersectionality, Screaming Silences, Black women's sexual and reproductive health, The Silences Framework, diaspora research, marginalized perspectives

## Abstract

Understanding the needs of Black women within a cultural and medical framework which recognizes the impact on health and well-being on the spaces where culture, health, and expectation intersect remains a challenge. In the UK, Black women are often more likely to have poor prognosis, worse outcomes and greater morbidity from treatable and preventable health conditions than their white peers. UK researchers have struggled to find a culturally appropriate safe methodological framework to help explore the challenges faced by Black women and their families in safeguarding their health, particularly around sensitive issues such as sexual and reproductive health. This article presents a relatively new intersectional framework which has been use for conducting health research on culturally sensitive health issues. The Silences Framework introduces the notion of “Screaming Silences.” Screaming Silences (or Silences) reflect the unsaid or unshared aspects of how beliefs, values and experiences of (or about) some groups affect their health and life chances. The article will explore how, the framework aligns with existing Intersectional approaches and how it could be used to expose intersectional nature of issues which influence and inform both individual and group understandings Black Women's health using examples relating to sexual health and life chances for Black women in the Diaspora.

## Introduction

In the UK as elsewhere, inequity exists in relation to the health, well-being and life chances of people from different parts of society, particularly across racial and gender lines (Umberson et al., 2014; Brown et al., 2016; Assari et al., 2017). Furthermore, the historical, and currently increasing, movement of people across the globe both voluntarily (for work or social mobility) and involuntarily (as a result of war or conflict) has resulted in a situation where many health conditions and infectious diseases (such as HIV, Ebola, Dengue fever for example) previously confined to one part of the world appear as challenges in geographically distant countries (Moore, 2012; Kaplan and Ng, 2017). As such there is an increasing need to incorporate understandings of diasporic identities (i.e., the differing ways in which people with ancestry from the Africa) and global perspectives of health and well-being as a key part of twenty-first century healthcare and efforts to improve the well-being of communities (Chen et al., 2017; Pottie et al., 2017). Diasporic identities is taken in this context to refer to fluid and changing (rather than static) instances in which people with an ancestral or recent history of migration from outside their current place of residence, retain objective components of the territorial homeland, such as a shared history, language and culture (Christou and Mavroudi, 2016).

Black women living in the UK are reported as having poor prognosis, worse outcomes and greater morbidity from treatable and preventable health conditions such as Diabetes, cervical cancer and heart disease than their white British peers (Calabrese et al., 2015; DeSantis et al., 2016). In the face of such disparities in health outcomes, understanding the healthcare needs of Black women within a medical framework which recognizes how health and well-being are impacted by identities and experiences of marginalization becomes increasingly essential (Aquino et al., 2012; Haynes-Maslow et al., 2016). UK researchers to date have often struggled to find a “culturally safe” methodological framework to help explore the challenges faced by Black women and their families in safeguarding their health, particularly around sensitive issues such as sexual and reproductive health. In this context, by culturally safe, I mean approaches which centralize (Black women's) experiences in the spaces where identities, culture, health and expectation intersect and which the women themselves report as being appropriate and inclusive of their needs.

To date, health care focused sexual health research, while it has moved some way away from simply adopting an illness and disease based approach with the focus on infection identification; remains unable to adequately account for variations in health and life chances for some of the most disadvantaged groups in society (Williams and Mann, 2017). However, they have yet to find robust ways of facilitating ways of understanding or interrogating the personal, shared, and community based processes by which structural disadvantage intersect with social identities to produce lived experiences of exclusion and consequently poorer health outcomes. Researchers on health inequalities for example, continuously point to social variation in education, economy and access to appropriate healthcare and the associated socioeconomic disadvantage that results, as being at the heart of inequity in mental and physical health outcomes (Ferraro et al., 2016; Fong et al., 2019). However, while it is not always possible to quantify the impact absolutely, evidence of the consequences may be observed in poorer health outcomes and life expectancy in some of the most disadvantaged communities in society.

Conducting health research to measure, understand or evidence how exclusion or marginalization may impact on health outcomes is often complicated by historical, political and professional sensitivities around race and inclusion (Serrant-Green, 2011; Viruell-Fuentes et al., 2012; Nyashanu and Serrant, 2016), which in turn is further complicated in areas of sensitivity such as sexual health (Greaves, 2015). Lack of even a basic intersectional approach to health and well-being, one that takes into account the social, psychological and cultural influences on health and behavior (as well as the physical) continues to dominate health care policy and even health care practice. Consequently, the underpinning evidence base, driving health care service development and care decisions is developed without consideration of the complexities concerning how people, particularly those whose identities are racialized and genderized, live their lives. As a result of the lack of differentiation between the experiences of women for example, the experiences of some BME women still remain under researched, absent or rendered invisible as they are hidden within general reporting of statistical health data. This approach commonly has little or no discussion of how the clinical outcomes measured are impacted by or impacts on the broader aspects of women's lives and health. For example, while many women may experience menopausal symptoms, the cultural meanings ascribed to it by different ethnic groups, as either a natural phase or medical condition to be controlled, affects whether help is ought to manage symptoms, the nature of the help requested as well as the psychological impact on the woman of needing to seek out medical assistance.

A robust methodology for exploring marginalized (or hidden) experiences, particularly in relation to sensitive issues such as sexual health, is needed to facilitate our understanding of the parameters within which Black women's sexual health is constructed by healthcare providers and services and consequently how this impacts on their identities and understandings of self (Nyashanu and Serrant, 2016). This is important as sexual health decision making is recognized as being influenced by cultural and socioeconomic factors as much as medical knowledge or physical symptoms (Hankivsky, 2012). For example, researchers have reported how BME women (and men) accessing sexual health services and support often modify their pattern of service use, or acceptance of advice given, dependant on their perceptions of how clinical professionals view people from their particular ethnic group (Nyashanu and Serrant, 2016; Prather et al., 2016). In addition, they also, by association make “judgements” about themselves as individuals (Serrant-Green, 2004; Nyashanu and Serrant, 2016; Prather et al., 2016). Therefore, reliance solely on medical parameters or infection risk to inform clinical guidance makes it difficult for health care provider and professionals to effectively improve the sexual health of people from marginalized groups, or indeed where we should focus our efforts.

Intersectionality has developed over the last four decades as a sound basis for understanding multiple contexts of Black women lives as racialized and gendered subjects (Crenshaw, 1989; McCall, 2005; Collins, 2015a). However, researchers have highlighted the failure by many academics to date to sufficiently focus on developing methods and methodologies to guide research–especially in healthcare–with currently there being little real advance, particularly outside the USA on *use of* intersectionality as basis for analysis.

Through the exploration of the intersectional nature of Black Women's sexual and reproductive health in the UK as an example, I present here an emerging framework for conducting health research on culturally sensitive health issues, The Silences Framework (Serrant-Green, 2011) to address this need. The Silences Framework has been successfully used elsewhere for conducting research on sensitive issues from the perspective of individuals and groups whose lived experiences of health and well-being have previously remained “silenced” (Eshareturi et al., 2014, 2015; Nyashanu and Serrant, 2016; Janes et al., 2018). In this paper I will explore how the framework, may be aligned with, and drawing on aspects of existing intersectional theory and frameworks, can be used by researchers to expose the intersectional nature of issues which influence and may inform individual and group understandings of health, well-being and life chances for Black women in the UK diaspora.

## Locating Black Identities in the UK Diaspora

Prior to discussing the Silences Framework, it is important that the terms “Black women” and “diaspora,” as contested terms, be clearly articulated, as they will be used in this paper. In doing so I do not intend to present a critical discussion as to why the proposed definitions have been selected as this has been central to many scholarly outputs, particularly by Black feminist writers (Davis, 1993; Brah, 2005; Collins, 2015a). These are presented here in order to contextualize for the reader my working definition for use of term “Black” and which Black women are centralized in this discussion of Black women in the UK Diaspora.

The term Black is used in this paper as a political term to represent people in the UK who identify as being of African and African-Caribbean origin. It is used to reflect a unity of experience of racism, discrimination and prejudice in the UK often faced by people whose skin color is not white (Serrant-Green, 2002).

The term Diaspora has many uses and reference points (Hall, 2014; Ackah, 2016; Ndhlovu, 2016). In its truest sense, it is used as a term to denote the dispersion or spread of peoples from their original homeland (Oxford English Dictionary Online, 2018). In this paper, it is used to refer to peoples from the former British colonies, who now identify with communities, which form part of the current British Commonwealth countries. Migration and diasporic identities have informed the historical formulation of the United Kingdom and continue to shape its approach to diversity, inclusion and equality (Sökefeld, 2016; Chamberlain, 2017).

While economic migration between the UK, Africa and the Caribbean has to some extent occurred throughout history, the mass migration as a result of “invitation” from the UK itself started in earnest in the 1950s and peaked around the late 1960s (Harper and Constantine, 2010). Continents like Africa and the Islands of the Caribbean are geographically distinct from each other, with their own cultural identities, social norm and language. However, they retain the shared experience of migration (for educational, economic, and familial reasons), historical links to colonialism, slavery trading and control by Western countries (in this case the UK) (Hall, 2014; Bolton, 2015; De Goede, 2017). Successively therefore the UK has seen, and benefitted from developing and emerging diasporic identities with each generation of “migrants” together with their descendants contributing to increasingly complex associations with “homeland,” nationality and experiences of “belonging” (Hall, 2014; Crosbie and Hampton, 2015).

Statistically in the UK, according to the 2011 census, ~11% of people living in the UK identify themselves as being from Black minority ethnic communities. The categories themselves are complicated by a lack of clear definitions, assimilation of many groups differing ethnic categories in some, and finally by the self-determination of category (where people identify for themselves which group they assign to). That being said, the largest group are reported as being Asian or British Asian (6.9%) followed by African, Caribbean or Black British (3.0%) with those identifying themselves as of mixed or dual or multiple heritage being the fastest growing group, currently ~2% of the population. Black women comprise 50% of the South Asian and slightly higher 52% of Black Caribbean/African populations (Office for National Statistics, 2012). It is important to have some indication as to the nature of the population under study in any research project–this is part of the first stage of The Silences Framework (see later in this paper) in setting the context for the study. The profile of the particular UK based minority ethnic communities detailed above identifies more than simply the growing numbers of people of Caribbean and African descent–it also highlights the complexities bound up in intergenerational differences in experiences as well as the nuances of multiple ethnic heritage as an increasing profile in the UK. All this points to the need for researchers to have at their disposal methodological approaches which are able to encompass changing, non-binary and nuanced identities without having to reduce them or confine them within restrictive singular categories.

## The Silences Framework (Overview)

The Silences Framework (Serrant-Green, 2011) was originally developed from the earlier research which established the concept of Screaming Silences: This first research study explored the sexual health experiences of Black Caribbean men and how perceptions of their sexual identities impacted on their sexual health decision making and use of sexual health services (Serrant-Green, 2004). Screaming Silences is a term used to define areas of research and experience which are little research understood or silences (Serrant-Green, 2004, p. 2). In essence, Screaming Silences often embody the voices of marginalized groups and experiences or viewpoints which are seldom evidenced in mainstream literature or readily available academic search engines.

The Silences Framework which is built around this concept was developed to provide a theoretical framework for researchers seeking to undertake research on “sensitive” issues from the perspective of marginalized research subjects. The theoretical approaches underpinning the original development of framework have much in common with feminist, anti-racist and critical theorist researchers (such as Smith, 1983, 1989; Hooks, 2000; King, 2016), while not necessarily remaining true to one. The core premises of The Silences Framework (as with other anti-essentialist approaches) are that

- Research and experience are both context bound;
- Inequality and socially assigned power impact on experiences of people in society;
- The researcher plays central role at all stages, from determining what is researched through to affecting what or how evidence is produced and
- Finally and perhaps most importantly, it equal importance is placed on marginalized views and personal experience as on “expert” opinion.

The original 4-stage framework and its development has been discussed in detail elsewhere (Serrant-Green, 2011) and it is not my intention to do so again. However, in order to underpin this article it is important to note that the key principles at the heart The Silences Framework are to recognize and expose some of the inherent tensions in researching sensitive issues and “marginalized perspectives.” In doing so it also acknowledges the central role played by learned or assigned social scripts on health and life chances of an individual or their community. In essence, the aim in completing the four stages of the framework is to enable the researcher to unite the “known” (what is evidenced and previously reported) and “unknown” (the silent, little researched or hidden aspects of marginalized experiences) in order to bring about greater understanding and ultimately, inform change.

The Silences Framework has been used in a range of empirical studies, which centralize previously hidden or marginalized aspects of human experience in specific contexts that are deemed “sensitive.” These have included studies of experiences of health provision for newly released offenders (Eshareturi et al., 2015; Eshareturi et al., 2014), exploration of HIV stigma within Black Sub-Saharan African communities residing in the UK (Nyashanu and Serrant, 2016), living with comorbidities of HIV and Tuberculosis in Brazil (Rossetto et al., 2018) and the recovery experiences of young adults following proximal fracture of the femur following a low velocity fall (Janes et al., 2018) with further studies currently underway.

Empirical use of the framework by other researchers in studies beyond Black Caribbean men's sexual and reproductive health has informed this paper and for me, (re)thinking of the ways in which The Silences framework may be further enhanced in specific studies through association with other established theoretical approaches to engaging marginalized discourses such as intersectionality.

## Intersectionality and Researching “Silences”

Intersectionality can be described an analytical framework which challenges the notion that multiple social stratifications of power exist (or can be studied) as independent entities. It is based on a belief that socially determined categories have an element of interdependence and inter “activity” in peoples' lives, which can potentially both cause and increase marginalization in a given society. Through using intersectional approaches we can explore how the different aspects of people's lives (e.g., social, economic, educational, cultural) relate to each other and how they are positioned in hierarchies of power in a given society i.e., the importance placed on them relative to each other. This enables us to critically assess the resultant impact on people's access to resources and services within their communities and in turn, the impact of these “intersectional” aspects of living in society on the life chances of the most marginalized in society.

While originally coined by Crenshaw (1989) as an analytical approach, the underpinning premise of intersectionality has a long history in women's writings spanning the work of Black feminist writers in particular (see Kelly, 2009; Collins, 2015a for example). Over the last few decades, the original theoretical approach has been extended to apply beyond race and gender, to include a broader range of social boundaries and spaces (Kelly, 2009; Viruell-Fuentes et al., 2012). This has led to growing interest in variations of experience within marginalized groups as well as how they are differentiated from the majority or dominant group members which has given rise to studies investigating and seeking to understand the multiple notions of oppression and how they are experienced “intragroup” (McCall, 2005). In turning the attention of intersectionality toward “intra group” diversity of experience, McCall discusses the social, structural, and political hierarchies (such as education, poverty, or even cultural values) modify and create distinctions in the experiences of women in the same ethnic groups. This is a move away from the traditional gaze of studies around inequality and difference which predominantly focus on difference or variations in experience between different ethnic or cultural groups–what McCall Calls Inter-categorical (or inter-group) complexity (McCall, 2005). In contrast McCall highlights the requirement for researchers to include wider social determinants alongside race and gender including class, sexual orientation and even education in order to better understand experiences within a particular social and political context. If considered in relation to definitions of “Black” women (as mentioned above) this invites researchers to consider the ways in which the differing self-definitions of Black womanhood are impacted by or “intersect” within their particular social contexts resulting in variations between women in the same groups in terms of what it means to be “Black” African or African Caribbean in the UK.

Through successive developments, feminist theorist such as Smith (1983); Collins (1986, 2015b); McCall (2005) and others have reinforced the need in intersectionality to focus on an individual's (or groups) unique world view and use this as a basis to challenge the taken for granted beliefs about the world and push for change. Hence in the twenty-first century we sit at a juncture where intersectionality is being increasingly applied within a health context where gender, race, class, sexuality, and ability to access services intersect and are impacted by established policies, procedures and practices. In health care research, intersectionality has been broadly used to encourage the inclusion of different (seldom heard) voices or experiences in research to inform service developments and health care policy (Kelly, 2009, Viruell-Fuentes et al., 2012). These approaches provide a platform for challenging the established and often entrenched or dominant “medical model” of research and decision making which often functions on the basis of physiological modes of understanding health and illness. Researchers have argued that where service providers fail to take into account the broader range of factors that impact on health and decision making, provisions made to improve health and sustain well-being are less useful to some segments, usually those most disadvantaged in society (Cochran and Mays, 2016). Researchers incorporating intersectional approaches focus on including diverse views and experiences in studies in order to better understand why variations in experience exists and how best to account for these in order to improve health outcomes (Kelly, 2009; King, 2016).

However, despite increasing developments in intersectional theory there has been relatively slower progress in developing new methodologies in the form of processes and frameworks to study it (McCall, 2005). Researchers have previously identified that to some extent, the very nature of intersectionality, and its introduction of multiple dimensions of the world, brings with it, difficulties as to how to evidence or include these varied and varying aspects of experience in research studies or indeed how to approach it (Nash, 2008). Critics of intersectionality have highlighted three areas of challenge for researchers seeking to conduct studies, namely:

- Accounting for both shared experiences of disadvantage as well as individual (intragroup) variations.
- Enabling investigations to include more than race and gender (assigned characteristics).
- Achieving praxis (practical application).

This has resulted in calls and emerging studies to develop new methodological approaches which address these challenges by dealing with the complexities of how life is managed and lived in the real world. In doing so, such frameworks and processes work to facilitate the representation of diverse experiences in studies which are central to intersectionality.

The Silences Framework is presented here as an approach which supports researchers seeking to use an intersectional approach in their studies. It does this by reflecting the manner in which people are socially positioned within a given society, at a point in time and how they are engaged with the world (and with others) in a named situation or context. In this way The Silences Framework seeks to link theory (what should be) with everyday practice (what is) at a specified time point. In doing so, it aligns with McCall's view of intersectionality as aiming to study “relationships between multiple dimensions and modalities of social relations and subject formations” (McCall, 2005, p. 1771). The Silences Framework is conceived as being particularly suited to health related research where the subject under study is deemed to have a degree of “sensitivity” and the people at the center of the study are members of a marginalized group (Serrant-Green, 2011).

In the remainder of this paper I will use the four stages of The Silences Framework to illustrate how, it can be used to expose the intersectional nature of everyday experiences which influence and inform both individual and group understandings of the world. Examples related to studying the sexual health of Black women in the from African and African Caribbean communities in the UK Diaspora, as defined earlier, will be used to illustrate the potential benefits of using the framework.

## Stage 1: Working in “Silences”: (Contextualization)

The first stage of the Silences framework sets out to establish a contextualized, situated exploration of the research subject, illustrating how it is currently understood and represented in society. The aim is to set any proposed study in the “real world” of the participants from the outset i.e., the current social, political and cultural context of the society they inhabit to ensure that the “known” foundations on which a study is premised are presented. “Known” foundations include the information readily available in the form of traditional book, papers and reports as well as other alternative, more “silent” ways of knowing through “grey” literature and online sources. At the same time, this stage works to begin questioning *what is known about a subject and how that information is acquired*. In turn, this is used to begin to illustrate the impact of both the subject in question and the processes used to gather and verify the information on people's lives (and health). In essence, it presents our view of the world and the Screaming Silences (aspects that are less likely to be highlighted, researched or deemed by those in power as credible) retained in it. Through this raising of questions we begin to get a sense of the possible “gains” in conducting the study and why this subject needs to be studied or re-examined *at this time*: In health research this means setting out the importance of the study for care practice or optimizing the health of individuals or groups.

In a traditional sense Stage 1 of The Silences Framework incorporates the literature review phase of many studies. However, the need to include and value personal experiences, particularly those of marginalized and hidden voices means researchers have to go beyond published academic sources to include other “non-academic” or non-research based sources which more accurately represent diverse experiences. This aligns closely with the ethos of intersectionality in as highlighted by Cole (2009) in valuing personal experiences alongside the established theoretical debates often seen in academia and certified sources. In line with Cole and others, this first stage of the Silences Framework calls for the researcher to not only review the evidence as presented but to interrogate the structures or assumptions surrounding the information itself (Cole, 2009; Hankivsky, 2012; Viruell-Fuentes et al., 2012). The invitation to review the information provided in relation to process as well as content can be facilitated by applying Cole's questions “*Who is included within this category? What role does inequality play? Where are there similarities?”* (p. 171) Through this approach, wider evidence is produced to expose the pre-existing the social, political, psychological or clinical arena of the research while reflecting the complexities in the social world and challenging the limitations of the dominant (take for granted) viewpoints. In this way, this first stage of The Silences Framework and my development of it mirrors the work of Black feminists of the past (Collins, 1986; Hooks, 2000) in that it encourages researchers to consistently seek ways of engaging with assigned social categories while taking a more critical approach to them. The overall efforts are focussed on inviting active, structured interrogation of the social categories themselves and the personal embodiment of in the lives of the people involved through their different experiences.

In the context of Black Women's sexual health and diasporic identities this first stage illustrates how Black women are positioned in society in relation to their sexual health and the ways in which Black women's identities and life chances are impacted by that positioning. For example, the current context in which variations exist in how Black women from African Caribbean and African communities are perceived as more or less (Hyper) sexualized in UK society and how this is played out through their experiences of using sexual health services, or making sense of their own sexuality is key (Carby, 1985; Daniels, 2016; Rosenthal and Lobel, 2016). If researchers fail to take into account the context and processes surrounding how Black women are positioned in relation to their sexuality by for example, adopting an intersectional approach utilizing The Silences Framework, studies may fail to reflect the true nature of what happens to Black Women in the real world. Instead The Framework in tasking the researcher to contextualize the “known” and “silenced” areas of knowledge about the sexual health encourages a move beyond the quantitative surveys which often dominate sexual health toward broader categories of supporting evidence to consider differential but socially important variations in sexual health experience at the intersections of race, class, education and gender. In this way, using The Silences Framework researchers are able to critique and question past approaches to sexual health while remaining sited on the reality, impacts and modifying effects of power within prior categorizations on Black women and their sexual health. This enables simultaneous consideration of theory and practice relating to Black women's multiple subordinate positions without the need to present a fragmented representation of each separate category as has occurred in past studies (Daniels, 2016; Rosenthal and Lobel, 2016).

## Stage 2: Hearing “Silences”: (Location)

This next phase of the framework focusses on locating or “exposing” the Silences inherent in conducting *this research study, by this researcher, at this time*. This stage includes reflection on a tripartite focus, which recognizes the existence and importance of the dynamic relationship between researcher/research subject/participants in a study. The Silences Framework operationalizes central aspects of criticalist approaches, which have challenging markers of race, gender or other socially assigned power dynamics at their heart (Collins, 1986; Agger, 2006; King, 2016). In criticalist approaches such as feminist, ethnicities or sexualities based research the nature of the relationship between researcher/research subject/participants shapes both the study itself and the evidence produced as a result (Serrant-Green, 2011). This stage therefore identifies the “Silences” (unknown or seldom discussed) aspects of the tripartite relationship, which may impact on the research through critical discussion of the social assigned categories and the relevant intersectional aspects of experience housed in the researcher identity (who is conducting the study), research subject (what is being studied) and participants (who is being studied). In exploring these aspects of the research study, The Silences Framework follows the underpinning assumption of intersectionality as described by McCall (2005) in asserting that complexities and contradictions of knowledge take place within the subjects as much as the external context. Following the exposure of these “silences” considerations for accessing participants by the researcher, phrasing of questions or even what ethnic or cultural sensitivities there may be around discussing the subject itself, are revealed which, in the next phases to come, will help provide the rationale for data collection, analysis and subsequent discussion. In pre-empting as far as possible, these methodological considerations in advance, further context for the research to be undertaken is established ensuring that the research design and subsequent findings can be understood in the light of these Silences rather than despite them (Serrant-Green, 2011).

## Stage 3: Voicing “Silences”: (Verbalisation)

The third stage of The Silences Framework incorporates the data collection phase of the research study. Here the researcher explores the situated views and experiences of marginalized individuals and communities living within the silent spaces at the center of the research. This phase which epitomizes the approach to data collection is focussed on encouraging verbalization of what it is like to live in the identified silent spaces in the study and seeking to redress the balance of “whose” voices are heard or prioritized as conduits of personal stories through adopting a philosophy of “speaking for ourselves.” As such, the marginalized (silent or silenced) voices and user perspectives, which are the central tenet of the study, must be included–The Silences Framework and its uses are built around this as a key aspect of feminist praxis (Hooks, 2000; King, 2016). In doing this–the intersectional realities of living with, within and between socially assigned categories in positions of silence, marginalization or invisibility are presented and shared from the center. In relation to Black women's sexual health for example, incorporating Black women's experiences of using sexual health services, allows the differential aspects of how one service operates toward one group of Black women alongside another to be heard. This is facilitated though exposing the similarities and differences arising from the personal and contextual aspects of Black women's experiences and their relationships with the assumptions made externally relating to relative “risk” and those made internally (within group) about the acceptability of utilizing the service by Black women themselves. The Silences Framework, used in this context would allow us to hear and witness women's first hand experiences in its complexity–reducing the need to fragment and thus further silence what are very human experiences in order to understand them. In the studies utilizing the Silences Framework, the voices heard are real, often raw but always firmly located in “what is” for people occupying these spaces (Nyashanu and Serrant, 2016; Janes et al., 2018).

The exact methods used to gather the data are not specified in The Silences Framework, as these are determined to a great extent by the question under study as well as the outcome of the exploration of the inherent Silences in the study conducted in Stage 2. In line with other inclusive approaches to generating evidence, this stage of The Silences Framework also houses an intersectional approach to analysis, which encourages the researcher to critically reflect on a broad range of issues arising from their own initial analysis of the data. It does this by requiring the researcher to recognize the influence of group or shared understandings, alongside individual participant positioning or experience, on aspects of identity formation and health behaviors. A phased, reflective and cyclical approach to the process of data analysis is therefore included in The Silences Framework, which acknowledges and aims to counteract the possible limiting effects of single researcher analysis on outcomes. The Silences Framework, unlike other approaches, therefore requests that researcher goes beyond simply “sense checking” their analysis only with participants or another “expert researcher.” Here the researcher needs to also seek out the “collective voices” of those in the wider but linked social networks related to the participants or research subject in helping to shape, inform and challenge their own “researcher perspective” during progressive stages of analysis. For example, in researching black women's sexual health, the researcher would be required to seek critical comment on their initial phases of analysis from sexual health professionals, other Black women on included in the sample and even partners of Black women–all of whom are part of the collective knowledge (voices) that make up the social network impacting on Black women's experiences in using sexual health services. This not only helps to re-affirm the validity and reliability of the data analysis but also ensures that any related concerns which may possibly not have been raised by many participants have an opportunity to be explored or provide the researcher with additional “prompts” to revisit their initial analysis so that vital “silences” are not missed.

## Stage 4: Working With “Silences” (Re-Contextualization and Discussion)

The final stage of The Silences Framework ensures that the practical intent or “usefulness” of findings from studies using this approach is not lost in the process of completing the study. Here, the possible impact of the findings on the current context as presented in Stage 1 are discussed in the light of the findings from stage 3 and of equal importance the “silences”(new or otherwise) arising out of the study itself, are exposed. The key question is “so what?”–How has world changed as a result of this study? What is the value has the inclusion on marginalized perspectives added to our understanding of the subject or the pre-existing context identified in stage 1—what distance have we traveled, and in which direction?

In essence, stage 4 of the framework requires a re-contextualization of the findings producing a “real world” discussion as final stage of study. It therefore includes detailed reflection on the theoretical contribution and pragmatic gains arising out of study outputs. However, the recognition of the impact and influence of power dynamics on persons at the center of studies using this approach means that decisions of what to report and how bring with them a degree of social risk and responsibility for the researcher. The very fact that the voices included in studies are often marginalized or silenced in society means that the decision of what to report also requires critical reflection and consideration of the impact on people and communities. It is unlikely for example that findings from a study exploring the impact of HIV stigma on Black Sub-Saharan African women (Nyashanu and Serrant, 2016) will bring about immediate changes in gender power base within the local communities involved. In addition, during the verbalization of experiences at sage 3, many women indicated verbally and otherwise some of their concerns about what it means to “speak out” from within their communities—this meant the researcher had to consider the future cultural acceptability for the women of what would be reported as well as the “ethical” issues relating to measurable risk.

As with all intersectional approaches The Silences Framework recognizes the dynamic nature of socially assigned categories and their ability to shape and impact the self-agency of individuals (Serrant-Green, 2011; Bauer, 2014). In The Silences Framework, the changing nature of inclusion, identity and belonging is recognized as being evidenced through the Silences surrounding many experiences in Black women lives. This can particularly be seen in the ways Black women negotiate changing expectations or representations of them in society and the ways these changes impact on their choices and life chances. One of the central tenets in adopting The Silences Framework is that the researcher themselves are recognized as part of the process of framing the participants' stories revealed through use of the framework. This in turn raises the issue that in conducting research, further silences may be created as well as unearthed through the research process (Eshareturi et al., 2015). The recognition of the “wholeness” of an individual through an intersectional approach using The Silences Framework i.e., the fact we cannot separate the different aspects of Black female sexual identity and hope to accurately reflect the voices of Black women talking about their sexual health—means that in the final critical discussion (Stage 4) and re-contextualization of the findings is more than a summary of what was discovered and discussed by participants; but a presentation of “what can now be said” about the subject; and by association, what still remains hidden (rather than undiscovered) as a result.

## Concluding Section

This paper has presented a relatively new theoretical framework, The Silences Framework as a possible option for applying Intersectional approaches to the research process. It is particularly suited (but not exclusively) for researching “sensitive” issues from the perspective of marginalized subjects. It is built on a concept called Screaming Silences which is closely aligned with the fundamental approaches to intersectionality that take time, space as well as social categories into account (McCall, 2005; Nash, 2008).

Screaming silences', like many aspects of any society are a product of the time spaces they occupy and the way in which the effects of power and inequality are experienced by an individual in a particular timeframe. ‘Screaming silences’ may be derived from, or illustrate, the ways in which power is used to determine an arbitrary norm at a particular historical and political point in a society

*(Serrant-Green*, *2011**)*

The Silences Framework, which arises from this concept, does not seek to present a critique of past approaches to intersectionality, nor is it blind to the reality impact or modifying effects of power on individuals or groups. Screaming Silences (or Silences) are identified as being situated in the personal and shared experiences of human beings and so as presented by McCall (2005) recognize the socially assigned categories which delineate “difference” in society (rather than rejecting them) but yet using them critically to help understand differing experiences. The Silences Framework embraces this working with and within “Silences” in order to expose them through the dynamic relationship between researcher, subject and research participants. In doing so it provides an answer to the call from proponents and critics of intersectionality as to the lack of a defined methodology and for development of new frameworks or research approaches which allow for both researcher and participant voices (McCall, 2005; Nash, 2008; Bauer, 2014) in seeking answers on the hidden, devalued or silenced areas of human experience.

By using The Silences Framework we have an opportunity to address some of challenges to intersectionality. In particular, the challenge of how we are able to pragmatically manage the range of different and often conflicting social categories embodied in one (Black) women. The “wholeness” approach of The Silences Framework, which seeks to identify and evidence the complexity of human experience through each of its four stages, enables simultaneous consideration of multiple subordinate positions rather than a fragmented representation of each. The framework supports the researcher in moving through a broader reflection and critical reflection of the reality of (women) occupying these spaces including a detailed approach to analysis incorporating the voices of the central characters and others that make up the social network in which they manage their lives in a specific context. Thus, complexity is explored as existing in the spaces between identities as much as in the categorical identities themselves (McCall, 2005).

In relation to health research, The Silences Framework presents us with an opportunity to apply intersectional approaches to consideration of factors impacting on health and well-being of Black women, where traditional research focussing on measuring unilateral relationships between health choices across multiple groups and clinical analytical categories remains the gold standard approach (Van Herk et al., 2011; Kaplan and El Khoury, 2017). The Silences Framework moves beyond this and offers an approach to exploring complexities *within and between* social groups, categories or both, challenging research where contributions to Black women's sexual health and diasporic identities are often omitted due to lack of “critical mass.” The framework is therefore offered here to researchers to focus their intersectional studies on a named social “group” or experience as a “snapshot” of society in time, to shape our questioning, thinking or development around “what is,” rather than seeking representation (proportional or otherwise) of a named phenomenon.

## Author Contributions

The author confirms being the sole contributor of this work and has approved it for publication.

### Conflict of Interest

The author declares that the research was conducted in the absence of any commercial or financial relationships that could be construed as a potential conflict of interest.
